# P-748. Chronic wounds and xylazine exposure among people who use drugs in Baltimore and Washington, DC: prevalence, preferences, and testing methods

**DOI:** 10.1093/ofid/ofaf695.959

**Published:** 2026-01-11

**Authors:** Edward C Traver, Onyinyechi Ogbumbadiugha-Weekes, Meredith Zoltick, Claire Tindula, Tina Liu, Sabina Ghale, Meghan Derenoncourt, Miriam Jones, Ashley Davis, Dorcas Salifu, Lydia Mitchell, Meghan F Anderson, Rahwa Eyasu, Emade Ebah, Elana S Rosenthal, Sarah Kattakuzhy

**Affiliations:** University of Maryland School of Medicine, Baltimore, MD; Institute of Human Virology, University of Maryland School of Medicine, Baltimore, Maryland; University of Maryland Baltimore - Institute of Human Virology, Baltimore, Maryland; University of Maryland School of Medicine, Baltimore, MD; Duke University School of Medicine, Durham, North Carolina; Institute of Human Virology/University of Maryland School of Medicine, Baltimore, Maryland; University of Maryland, Baltimore, Baltimore, Maryland; HIPS.org, Washington, District of Columbia; Institute for Human Virology (IHV), University of Maryland School of Medicine, Washington, District of Columbia; University of Maryland Baltimore - Institute of Human Virology, Baltimore, Maryland; University of Maryland School of Medicine, Baltimore, MD; University of Maryland, School of Medicine, Annandale, VA; Institute for Human Virology (IHV), University of Maryland School of Medicine, Washington, District of Columbia; Institute for Human Virology (IHV), University of Maryland School of Medicine, Washington, District of Columbia; Institute for Human Virology (IHV), University of Maryland School of Medicine, Washington, District of Columbia; Institute for Human Virology (IHV), University of Maryland School of Medicine, Washington, District of Columbia

## Abstract

**Background:**

Chronic wounds in people who use drugs (PWUD) are frequently infected and may progress to severe, life-threatening infections. Such wounds may be caused by xylazine, a non-opioid adulterant of illicit fentanyl and avoidance of xylazine may decrease wound incidence and infection. Xylazine test strips (XTS, BTNX Inc.) are commercially available to check illicit drugs for xylazine, but more data is needed on xylazine prevalence and knowledge among PWUD, and it is unknown if XTS can be used to detect xylazine in urine.

**Table 1:**
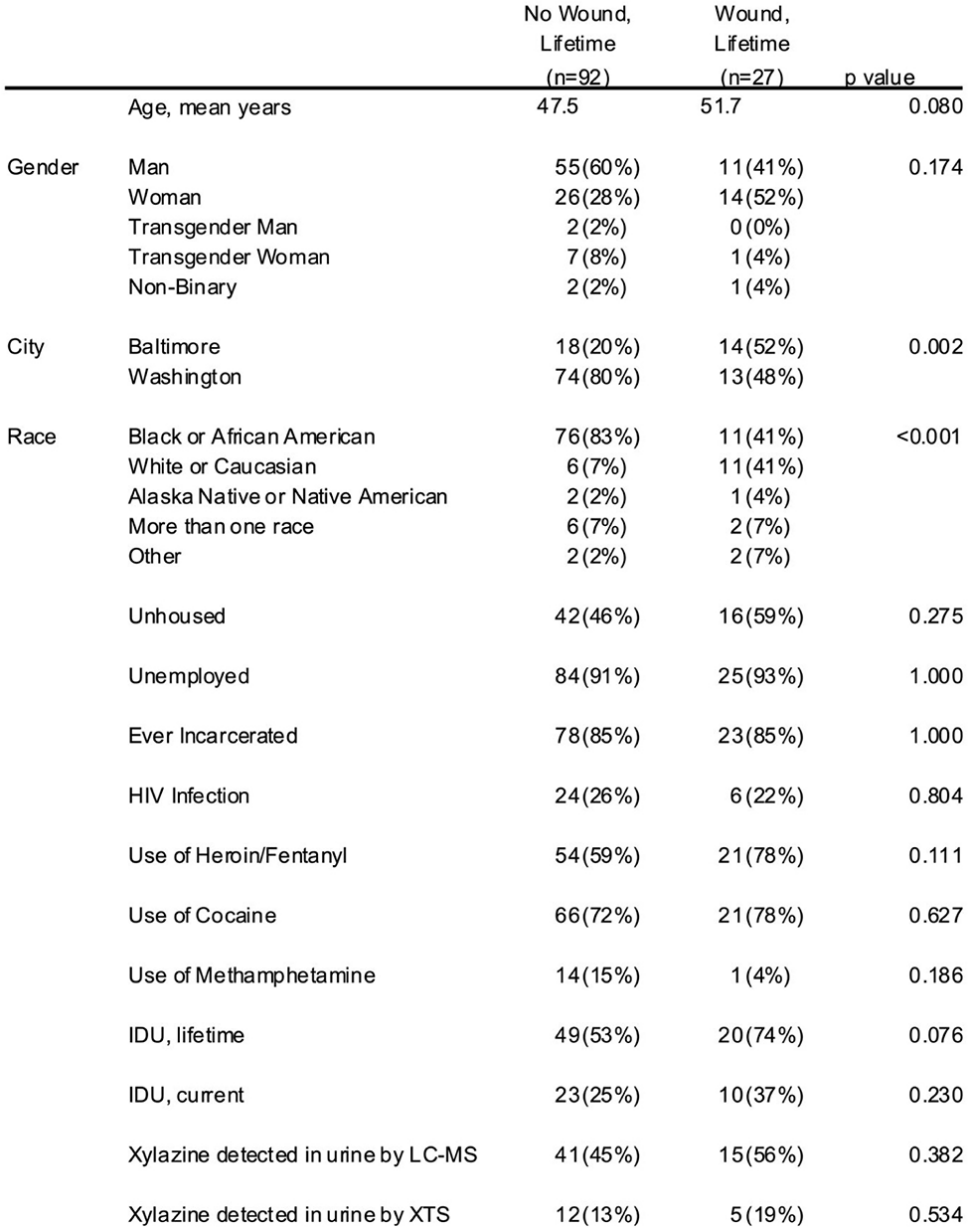
Demographics and Risk Factors for Lifetime Wounds. IDU, injection drug use; LC-MS, liquid chromatography-mass spectroscopy; XTS, xylazine test strip.

**Table 2:**
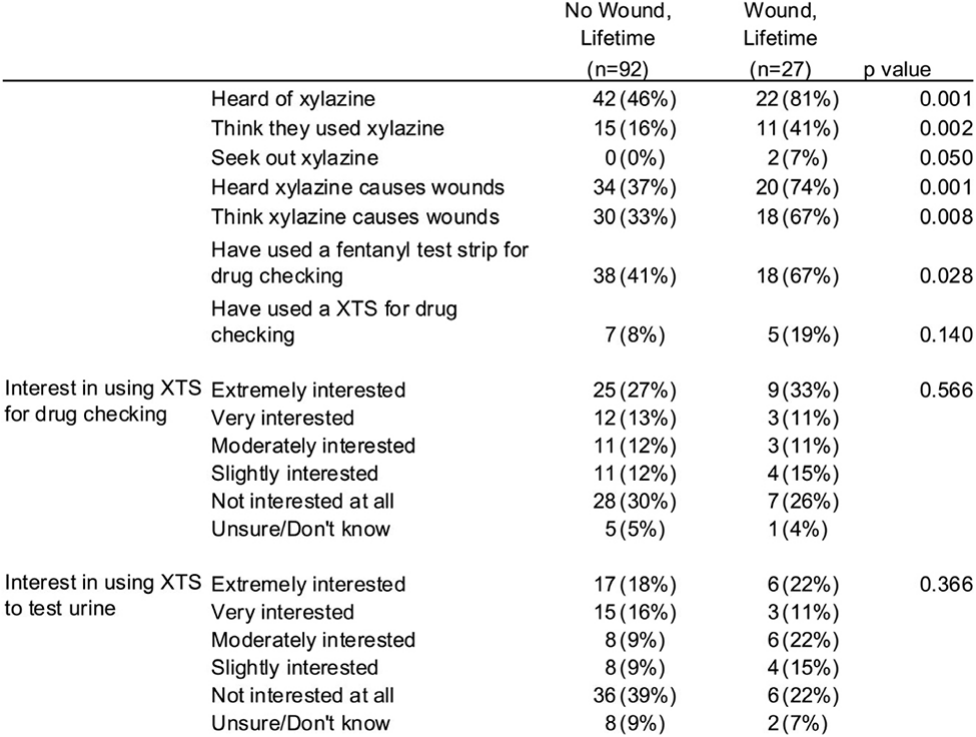
Knowledge and Attitudes towards Xylazine, Wounds, Drug Checking, and Urine Xylazine Testing.

**Methods:**

We surveyed patients at two clinics in Baltimore and Washington, DC that provide multidisciplinary care to PWUD. Patients were included if they reported non-prescribed opioids, cocaine, or methamphetamine in the past 30 days. Urine was tested for xylazine with XTS and standard liquid chromatography-mass spectroscopy (LC-MS). We measured associations between wound prevalence, xylazine urine positivity, and other demographic and clinical factors with Fischer’s exact test for categorical variables and Mann Whitney for continuous variables. We estimated the sensitivity and specificity of XTS to detect xylazine in urine compared to LC-MS and calculated 95% confidence intervals with the Wilson-Brown method.

**Figure 1.**
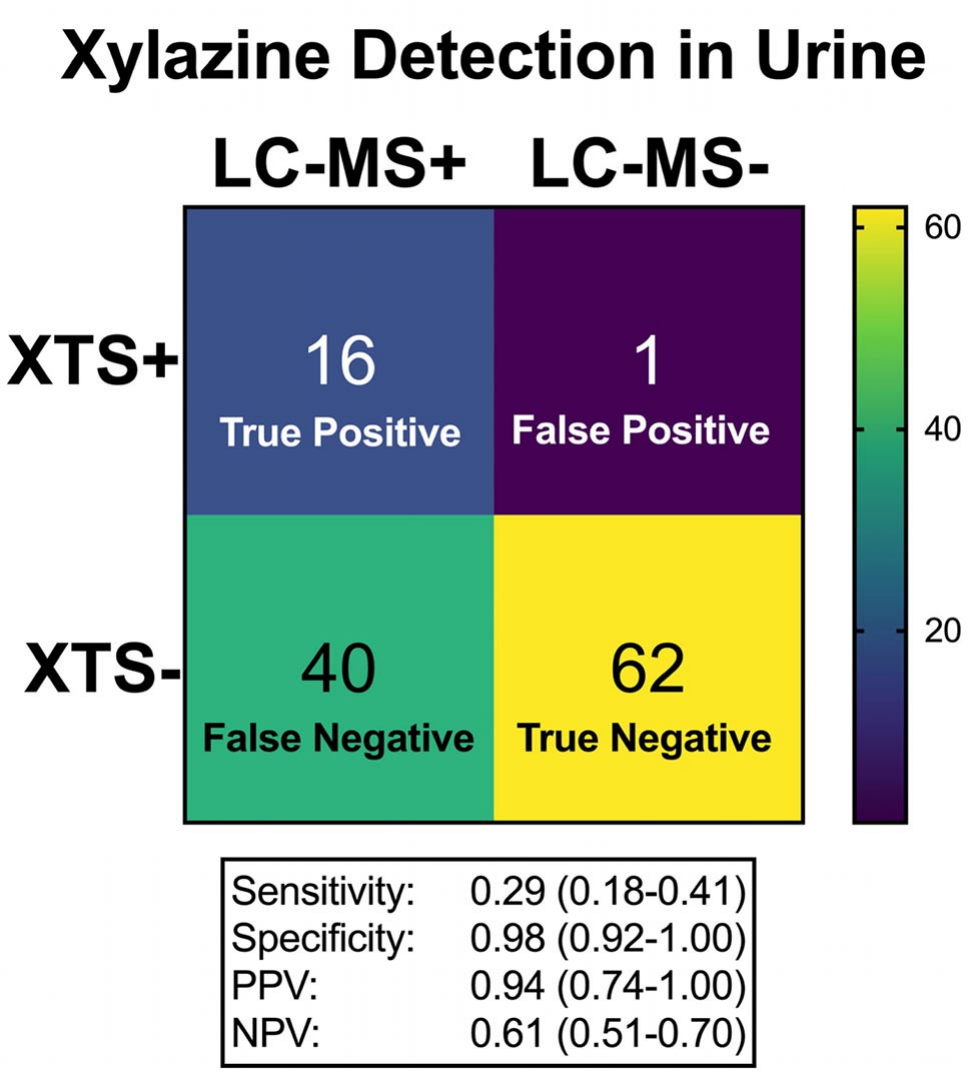
Figure 1. Test Performance of Xylazine Test Strip (XTS) compared to Liquid Chromatography-Mass Spectrometry (LC-MS) for Detection of Xylazine in the Urine. PPV, positive predictive value; NPV, negative predictive value.

**Results:**

119 participants were included; 27 (23%) who had ever had a wound (Table 1). Xylazine was detected by LC-MS in urine from 56 (47%) participants. People with wounds were more likely to be recruited from Baltimore (p=.002) and White or Caucasian race (p< .001). Wounds were not associated with injection drug use or xylazine detection in urine. People with wounds were more likely to be knowledgeable about xylazine (Table 2). XTS had a low sensitivity but high specificity for urine xylazine detection compared to LC-MS (Figure 1).

**Conclusion:**

Xylazine exposure at a single timepoint in PWUD in Baltimore and Washington was common but not associated with lifetime history of wounds, potentially due to variable exposure and detection over time. People with wounds were more familiar with xylazine. PWUD are interested in using XTS but the vast majority have not, suggesting residual structural barriers. XTS are have useful positive predictive value but low negative predictive value when used on urine.

**Disclosures:**

All Authors: No reported disclosures

